# Metal-oxide phase transition of platinum nanocatalyst below fuel cell open-circuit voltage

**DOI:** 10.1038/s41467-024-55299-3

**Published:** 2025-01-22

**Authors:** Carlos A. Campos-Roldán, Amir Gasmi, Meryem Ennaji, Morgane Stodel, Isaac Martens, Jean-Sébastien Filhol, Pierre-Yves Blanchard, Sara Cavaliere, Deborah Jones, Jakub Drnec, Raphaël Chattot

**Affiliations:** 1 ICGM, Univ. Montpellier, CNRS, ENSCM, 34095, Montpellier, France; 2 CIRIMAT, Université Toulouse 3 Paul Sabatier, Toulouse INP, CNRS, Université de Toulouse, 118 Route de Narbonne, 31062, Toulouse, France; 3 ESRF, The European Synchrotron Radiation Facility, 71 Avenue des Martyrs, CS40220, 38043, Grenoble, France

**Keywords:** Fuel cells, Electrocatalysis, Nanoparticles

## Abstract

The long-term stability of Pt-based catalysts is critical to the reliability of proton exchange membrane fuel cells (PEMFCs), and receives constant attention. However, the current knowledge of Pt oxidation is restricted to unrealistic PEMFC cathode environment or operation, which questions its practical relevance. Herein, Pt oxidation is investigated directly in a PEMFC with stroboscopic operando high energy X-ray scattering. The onset potential for phase transition of the nanoparticles surface from metallic to amorphous electrochemical oxide is observed far below previously reported values, and most importantly, below the open-circuit potential of PEMFC cathode. Such phase transition is shown to impact PEMFC performance and its role on Pt transient dissolution is verified by electrochemical on-line inductively coupled plasma mass spectrometry. By further demonstrating and resolving the limitations of currently employed accelerated stress test protocols in the light of metal-oxide phase transitions kinetics, this picture of Pt oxidation enables new mitigation strategies against PEMFC degradation.

## Abstract (web summary)

The stability of Pt-based catalysts is critical to the reliability of proton exchange membrane fuel cells. Here, the authors use stroboscopic operando high energy X-ray scattering to reveal Pt nanocatalysts experience a metal-oxide phase transition during conventional fuel cell operation.

## Introduction

The durability of proton exchange membrane fuel cells (PEMFCs) largely depends on the long-term stability of the costly cathodic platinum-based state-of-the-art electrocatalyst^1,2^. The highly corrosive environment of the cathode, where the oxygen reduction reaction (ORR) occurs, challenges the (electro)chemical stability range of most metals, even as noble as Pt^3^. This is especially true for nanoparticulate Pt under dynamic load profiles and is magnified during particular events such as hydrogen fuel starvation or during PEMFC start-up and shut-down. During such events, the cathode potential was shown to possibly exceed the open-circuit potential (OCP) of typically 1.0 V vs. the reversible hydrogen electrode (RHE), depending on the oxidant gas composition^4,5^. Tremendous research efforts have been dedicated to the elucidation of Pt catalyst degradation mechanisms above the OCP, which are now largely understood notably thanks to the recent development of in situ and operando characterisation techniques. Among them, studies on model Pt single crystal electrodes and commercial carbon-supported Pt electrocatalysts (Pt/C) using (surface) X-ray diffraction (XRD)^6–13^, and/or electrochemical on-line inductively coupled plasma mass spectrometry (ICP-MS)^12,14–18^ provided evidence for distinct mechanisms of Pt dissolution. Under dynamic potential (or load) profiles, Pt dissolution was found to be a transient process, triggered by Pt oxide-metal phase transitions (anodic dissolution during oxidation and cathodic dissolution during oxide reduction). A general observation is that significant transient Pt dissolution starts only at specific potentials above the OCP (ca. 1.05–1.10 V vs. RHE, depending on Pt surface structure). At such potentials, a phase transition from metallic to (amorphous) oxide occurs, which triggers surface Pt atoms extraction from their crystal lattice position by adsorbed oxygen species through the place-exchange mechanism^19^. Note ‘place-exchange’ is a historical term introduced in early seminal contribution^20^, but recent studies suggest that such extraction may eventually, but not necessarily (also depending on surface structure^7^), lead to complete Pt-O dipole inversion and the formation of subsurface oxygen at higher potentials. Also, this transformation of Pt atoms arrangement has to be distinguished from adsorbed oxygenated species on Pt steps already reported to occur at much lower electrode potentials^21^. Overall, it is considered from such observations that under conventional PEMFC operation (i.e., below the OCP), 4.8 nm Pt/C catalyst remains metallic (with adsorbed oxygen species) and should largely exceed 5000 h lifetime with only one Pt monolayer dissolved^22,23^. This is not, however, validated in practice^2^.

It must be stated that these conclusions regarding the onset potential for surface metal-oxide phase transition were derived from experiments strictly involving a combination of either (i) the use of model catalyst materials (single-crystal electrodes or thin-film catalyst layers), (ii) the use of an electrochemical environment different from that in a PEMFC (weakly-adsorbing aqueous electrolyte) and/or (iii) the use of unrealistic PEMFC operating conditions (such as room temperature or cathode potential range as large as 0.05–1.50 V vs. RHE). It is thus still largely unclear how this translates to the practical PEMFC during most of its lifetime, i.e., for cathode potentials below or at the OCP. In fact, few studies conducted on Pt nanoalloys report the early detection of Pt oxidation and/or dissolution at electrode potential close to the OCP, especially when outside of ambient conditions^10,24–29^.

Herein, the ability of high energy wide-angle X-ray scattering (WAXS) in revealing nanocatalyst oxidation trends operando^10,30,31^ is pushed significantly further by employing a stroboscopic measurement strategy (inspired from neutron scattering^32^) allowing the acquisition of ultra-fast data with high signal quality and temporal resolution (5 ms) in PEMFC. The observed trends in Pt oxidation, notably regarding the onset potential for surface phase transition from metallic to amorphous oxide, are supported by theoretical calculations and challenge the current understanding derived from model studies as well as control experiments performed on thin-film catalyst layers in 0.1 M HClO_4_ at room temperature (Supplementary Fig. S1). After establishing a correlation between early Pt oxide phase transition and overall PEMFC performance, the predicted transient Pt dissolution associated with the metal-oxide phase transitions is verified by electrochemical on-line ICP-MS experiments at 60 °C in 1.0 M HClO_4_ (Supplementary Fig. S2). Further analysis of the structural data provides first-order time constants for the oxide phase transitions in both model and PEMFC cathode conditions. In light of these time constants, the limitations of accelerated stress test protocols currently widely used for electrocatalyst materials stability screening are finally demonstrated by means of ultra-fast operando WAXS measurements. This picture of Pt oxidation occurring in PEMFC rationalises both its activity and stability limitations and enables new mitigation strategies against PEMFC degradation and the development of durable Pt-based PEMFC electrocatalysts.

## Results

**Fig. 1:**
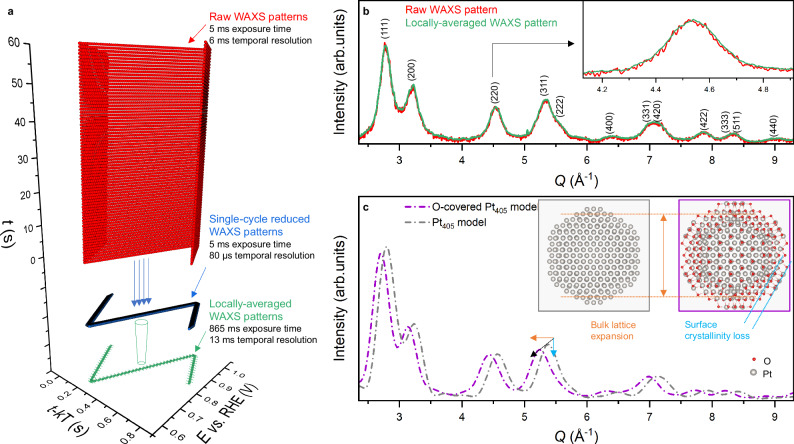
Stroboscopic operando WAXS to capture Pt cathode catalyst oxidation microstructural fingerprints with high resolution during (fast) potential cycling. **a** Exemplary 3D plot of 10,000 WAXS measurements (red markers) timestamp *t* as a function of the electrode potential and the period-corrected timestamp (*T* being the time period of a cycle and *k* the cycle number measured over 75 cycles between 0.60 and 1.0 V vs. RHE at 1000 mV s^−1^. Reduction of the 10,000 WAXS measurements in a single potential cycle (blue markers). Locally averaged WAXS patterns were obtained by applying a moving temporal average on the WAXS patterns (green markers). Errors bars relative to the potential and time values on the locally-averaged WAXS pattern symbols correspond to the standard deviations associated with the averaging process. **b** Signal-to-noise reduction associated with the increased exposure time on the locally-averaged vs. raw WAXS patterns plotted as a function of the momentum transfer *Q*. **c** Computed WAXS patterns of the 405-atom Pt nanoparticle models when Pt surface is free from adsorbates (grey) and when covered with oxygen (purple). The displayed two models of 405-atom Pt nanoparticle with optimised structures highlight the changes in bulk lattice constant expansion during adsorption of oxygen and surface crystallinity loss during metal-oxide phase transition, rationalising WAXS peaks shift (orange arrow) and intensity decrease (blue arrow), respectively. The PEM cell was operated at 80 °C with H_2_/N_2_ at anode/cathode, 104/400 sccm flow rates, 52% relative humidity and 100 mbar backpressure. The cathode potential is corrected from the cell's high-frequency resistance. Source data are provided as a Source Data file.

Figure 1 showcases the stroboscopic approach used here for operando WAXS, reasonably supposing the structural modifications are periodical for a short potential cycling duration. Taken as example the fastest measurement performed here (cyclic voltammetry between 0.60 and 1.0 V vs. RHE at 1000 mV s^−1^) in Fig. 1a, 10,000 WAXS patterns of the Pt/C catalyst collected over 75 potential cycles (5 ms exposure time, 6 ms temporal resolution) can be sequentially reduced in one single-cycle and locally-averaged to produce patterns reaching ca. 865 ms cumulated exposure time with only 13 ms temporal resolution. As shown in Fig. 1b, this operation largely improves the signal-to-noise ratio on the resulting WAXS patterns, allowing structural information to be obtained with much improved combined precision and temporal resolution on stable voltammograms (see Supplementary Fig. S3). This approach was used for all the operando WAXS measurements presented here, collected from either a commercial membrane electrode assembly (MEA, Fuel Cell Store) in an X-ray transparent PEMFC^33–36^ or a commercial Pt/C catalyst from Johnson Matthey (JM) in a thin-film electrochemical flow cell^30,31,37^ (later referred to as ‘model conditions’) for comparison. Transmission electron microscopy (TEM) images with associated particle size distribution of the two electrocatalysts investigated are available in Supplementary Fig. S4. The procedure was adapted to the different potential sweep rates used (see “Methods”).

As shown in Fig. 1c, and in agreement with previous reports^10,28,38–40^, the position, shape and intensity of Bragg reflections from metallic face-centred cubic (fcc) Pt phase are impacted by Pt surface oxidation, as rationalised here by 405-atom models of Pt nanoparticle. Despite the simplifying hypotheses on the nanoparticle environment and symmetry required for reaching practical computational time, the optimised structures (i.e., the position of each atom) when the Pt nanoparticle is free of adsorbate or covered with oxygen and their computed diffractograms predict qualitatively well the experimentally observed modification of the Bragg reflections during Pt oxidation (see in Supplementary Figs. S5, S6 and details about theoretical calculations in the Methods). Rietveld refinement of the WAXS patterns was used to extract the lattice constant, crystallite size and scattering intensity of the metallic Pt fcc phase as a function of the cathode potential (see Methods). Because electrochemical Pt oxidation is a surface process, its effect on the total scattered intensity is largely impacted by the nanoparticle size. For convenience, the scattering intensity was converted to surface crystallinity loss via normalising the scattering intensity by the surface atomic fraction derived from Pt nanoparticles’ size observed by TEM (see Supplementary Information). Complementarily, atomic pair distribution function (PDF) analysis was also performed complementary with the Rietveld method, to possibly gain further operando insights on the local structure of the formed Pt oxide phase (see “Methods” and Supplementary Fig. S7, S8).

**Fig. 2:**
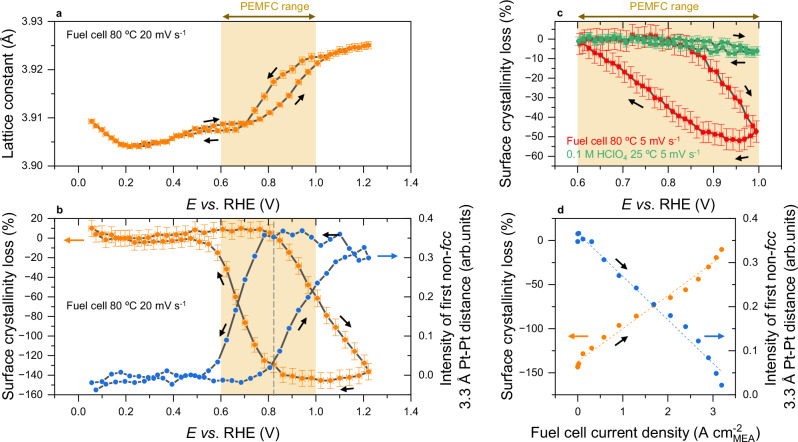
Revealing the critical potential for metal-oxide phase transition being at the centre of PEMFC cathode potential range. **a** Metal Pt fcc lattice constant variations and (**b**) surface-fraction-normalised scattering intensity variations of the Pt fcc phase and intensity of the first non-fcc Pt-Pt distance (3.3 Å) during cyclic voltammetry experiment in PEMFC between 0.05 and 1.23 V vs. RHE at 20 mV s^−1^. **c** Surface-fraction-normalised scattering intensity variations of Pt fcc phase during cyclic voltammetry experiment in PEMFC (red markers) and in electrochemical flow cell fed with 0.1 M HClO_4_ (green markers) between 0.60 and 1.0 V vs. RHE at 5 mV s^−^^1^. **d** Evolutions of the surface-fraction-normalised scattering intensity variations of the Pt fcc phase (orange) and intensity of the first 3.3 Å non-fcc Pt-Pt distance (blue) as a function of the fuel cell current density measured in a separated dedicated experiment (see Methods for fuel cell testing conditions). The orange boxes in (**a**, **c**) represent the PEMFC practical voltage range between 0.60 and 1.0 V vs. RHE, corresponding to the voltage of maximum power density and the open circuit voltage, respectively. In (**b**, **c**), the scattered intensity variations of the Pt fcc phase are normalised by their values at *E* = 0.4 V vs. RHE and *E* = 0.60 V vs. RHE, respectively. Errors bars correspond to the standard deviations associated with the refinements or computation of the different parameters. The X-ray transparent PEM cell was operated at 80 °C with H_2_/N_2_ at anode/cathode, 104/400 sccm flow rates, 52 % relative humidity and 100 mbar backpressure. The conventional cell was operated at 80 °C with H_2_/O_2_ at anode/cathode, 314/496 sccm flow rates, 100 % relative humidity and 500 mbar backpressure. The cathode potential is corrected from the cell's high-frequency resistance. Source data are provided as a Source Data file.

Using these microstructural fingerprints for Pt oxidation, different observations can be made from Fig. 2. Strikingly, the critical potential value triggering the metal-oxide phase transition is identified as being as low as 0.80 V vs. RHE from the onset of surface crystallinity loss (orange points in Fig. 2b). This is 300 mV below the generally accepted value of 1.10 V vs. RHE from model experiments on Pt(111) and 110 mV below a previous estimation from Sasaki et al. on Pt/C nanoparticles in liquid electrolyte^28^. Moreover, the onset of surface crystallinity loss is accompanied by the emergence of a non-fcc Pt-Pt distance of 3.3 Å (blue points in Fig. 2b), corresponding to a relaxed hexagonal Pt oxide layer relative to the ordered fcc phase according to Newton et al.^41^. Note both the onsets of crystallinity loss and non-fcc Pt-Pt distance are still 80 mV higher than the onset of lattice expansion (Fig. 2a), confirming the decoupling between early oxygen adsorption and the subsequent metal-oxide phase transition. Importantly, the onset potential for metal-oxide phase transition of 0.80 V vs. RHE falls right into the centre of the practical PEMFC cathode potential range i.e., between 0.60 and 1.0 V vs. RHE corresponding to the potential of maximum power density and the open circuit voltage, respectively (see “Methods” and Supplementary Fig. S9). In fact, a close comparison between operando WAXS data recorded in real PEMFC and in model conditions of the electrochemical flow cell (Fig. 2c) highlights the drastic differences between the two systems. Clearly, the data presented support the conclusions reached from previous model experiments regarding the rather low extent of phase-transition below 1.0 V vs. RHE in the diluted electrolyte at room temperature (ca. 7 % surface crystallinity loss), but the latter reaches ca. 50–60 % in PEMFC operated at 80 °C (Fig. 2b, c, depending on cathode’s history). The near-linear evolutions of the Pt fcc phase recovery (orange points) and decreasing intensity of the first 3.3 Å non-fcc Pt-Pt distance (blue points) as a function of the fuel cell current density measured in a separated dedicated experiment (see “Methods” for fuel cell testing conditions) in Fig. 2b strongly suggest that, under minimised/suppressed O_2_ mass transport limitation, such metal-oxide phase transition largely controls the PEMFC performance over the full range of operation. Finally, the observations made on the Pt/C catalyst largely transpose to a more advanced PtCo/C bimetallic catalyst (Supplementary Fig. S10), showing the broad significance and generality of the presented phenomenon.

**Fig. 3:**
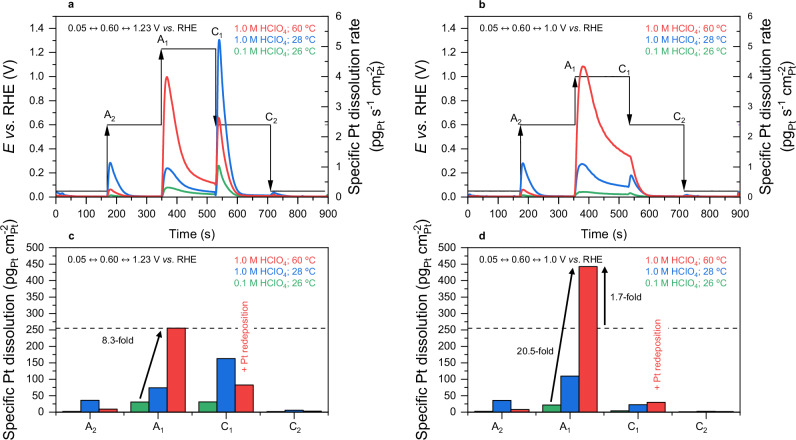
Transient specific Pt dissolution profiles from model to PEMFC-like conditions revealed by electrochemical on-line ICP-MS experiments with temperature control. Specific Pt dissolution profiles recorded (**a**) outside (0.60–1.23 V vs. RHE) and (**b**) inside (0.60 – 1.0 V vs. RHE) PEMFC cathode potential range during 3 min hold potential steps from 0.05 V vs. RHE under different conditions of electrolyte concentration (pH) and temperature. **c**, **d** Total dissolved Pt during the different anodic (A_x_) and cathodic (C_x_) peak contributions from panels (**a**, **b**), respectively. The ICP-MS measurements were performed on a commercial 40 wt.% Pt/C catalyst from Johnson Matthey (JM) and the electrode loading was 20 µg_Pt_ cm^−^^2^. Source data for panels (**a**, **b**) are provided as a Source Data file.

To confirm the effects of such early onset potential for metal-oxide phase transition on Pt transient dissolution in conditions relevant to PEMFC, specific Pt dissolution profiles from the commercial Pt/C JM catalyst were investigated by electrochemical online ICP-MS for different potential steps (3 min) under different electrolyte concentrations and temperatures (see “Methods”). The nature and balance of the different anodic and cathodic peaks displayed in Fig. 3a, b have been largely discussed previously in the literature^12,14–17^, and the observed increase of Pt anodic transient dissolution peak intensity (A_1_) as the electrolyte concentration and temperature increase was expected^25,42^. However, it is worth focusing on the comparison between potential steps performed within 0.05 $\leftrightarrow$ 0.60 $\leftrightarrow$ 1.23 V vs. RHE and 0.05 $\leftrightarrow$ 0.60 $\leftrightarrow$ 1.0 V vs. RHE. Contrary to the generally accepted trends in low-concentration electrolyte and at room temperature where A_1_ intensity decreases with the upper potential limit^43^ (also observed here), the opposite trend is found at higher electrolyte concentration and temperature. Quantitively, the integration of Pt anodic dissolution rates in Fig. 3c, d reveal the surface state reached at OCP conditions being nearly 2-fold more damaging to Pt compared to higher potential. In fact, a recent contribution from Fuchs et al. focusing on a Pt(100) extended surface^12^ further described the metal-oxide phase transition as a concomitant growth of two Pt surface oxide types. The anodic dissolution was attributed to the fast nucleation and growth of a first, stripe-like oxide in which extracted Pt is highly unstable against corrosion. The second disordered (hydro)oxide phase, on the contrary, was found to progressively passivate the surface and disable Pt anodic dissolution at higher potentials while controlling the cathodic dissolution during surface de-passivation. From this subtle interplay between these two oxide species formation, Pt(100) was found to be the most vulnerable to anodic dissolution at a potential of *E* = 1.2 V vs. RHE in model conditions. The present results indicate this critical potential is necessarily lower for Pt nanoparticles in a PEMFC. Note the associated cathodic dissolution rates C_1_ cannot be accurately estimated from online ICP-MS due to enhanced Pt redeposition kinetics at high temperatures, as suggested by previous reports^25,42^.

From combined operando WAXS and online ICP-MS experiments, it is clear that the early onset potential of metal-oxide phase transition occurring in PEMFC must largely account for both Pt-based catalyst activity and stability limitations. To further guide new mitigation strategies for catalyst degradation, structural data (surface crystallinity loss) collected during potential steps were analysed to extract the amplitudes and first-order rate constants associated with the metal-oxide phase transitions below the OCP during Pt oxidation and reduction (see “Methods” Supplementary Fig. S11 and Supplementary Table S1).

**Fig. 4:**
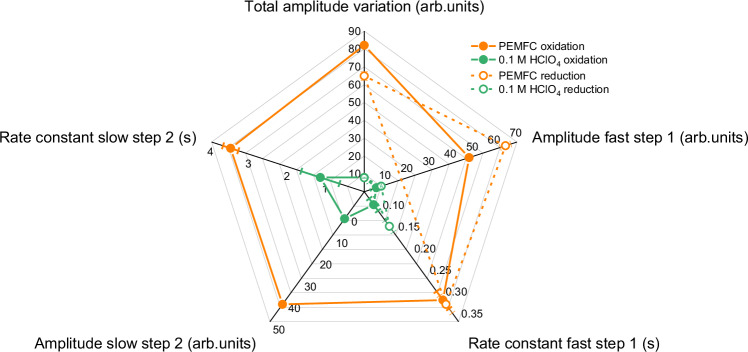
Determination of first-order rate constants and amplitudes associated with the metal-oxide phase transitions in both PEMFC and model electrochemical environments. Surface crystallinity loss from operando WAXS data recorded during potential steps between 0.60 to 1.0 V vs. RHE and 1.0 to 0.60 V vs. RHE were fitted using a linear combination of one or two exponential functions. Two functions were found necessary to capture two distinct steps during oxidation: a fast process (step 1) and a slow process (step 2). This was not found necessary during the reduction, which occurs in one single fast step (step 1). The radar chart allows easy comparison of the fitted parameters from one electrochemical environment to the other: the total amplitude variations of surface crystallinity loss also with the amplitudes and first-order rate constants associated with the different steps. The filled and hollow markers correspond to the oxidation and reduction data, respectively. The error bars represent the standard deviations associated with the fitted parameters.

As shown in Fig. 4, the Pt oxidation is divided into two steps (the fast and slow steps are labelled step 1 and step 2, respectively) featuring distinct rate constants, while the reduction proceeds in one single fast step. Note the presence of two steps during oxidation is in agreement with previous observations on nanoparticles from Sekizawa et al. for larger potential excursions, and the slow step is related to subsurface events such as the formation of subsurface oxygen^44^. In general, there is a near-constant 3.5-fold increase for both the amplitudes and rate constants of all steps of oxidation and reduction in PEMFC compared to model conditions. In fact, the rate constants for oxidation in PEMFC reach ca. 300 ms and 3.6 s for step 1 and step 2, respectively, both contributing equally to nearly 50% of the total amplitude of crystallinity loss. This means completing 95% of the second step of oxidation at OCP will take 3 times these values, i.e., more than 10 s. Note the slowest time constants extracted from the fits exceed the *RC* time constant^45^ of both electrochemical cells used in this work by at least one order of magnitude (see Supplementary Fig. S12 and Supplementary Table S2). Such slow temporal evolution questions the concept of accelerated stress tests (ASTs) routinely employed in the literature when screening material stability, as discussed below.

**Fig. 5:**
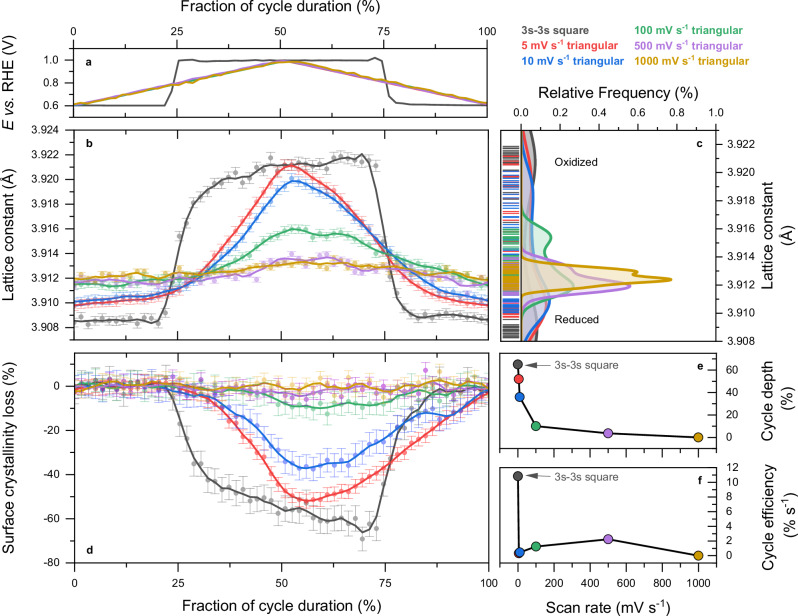
Outpacing Pt oxidation in fuel cell catalyst layer during accelerated stress tests. **a** Reconstructions of the applied potential cycle profiles between 0.60 and 1.0 V vs. RHE: 3 s – 3 s square wave and triangular waves at 5, 10, 100, 500 and 1000 mV s^−^^1^, (**b**) lattice constant variations with (**c**) associated Kernel density distribution. **d** surface-fraction-normalised scattering intensity variations. **e** Maximum of surface crystallinity loss induced by each potential profile type (cycle depth) and (**f**) rate of surface crystallinity loss induced by each potential cycle profile (cycle efficiency). The PEM cell was operated at 80 °C with H_2_/N_2_ at anode/cathode, 104/400 sccm flow rates, 52 % relative humidity and 100 mbar backpressure. The cathode potential is corrected from the cell's high-frequency resistance. Errors bars correspond to the standard deviations associated with the refinement or computation of the different parameters. Source data for panels (**b**, **d**) are provided as a Source Data file.

The full power of ultra-fast operando WAXS measurement was thus used to benchmark Pt oxidation trends during fast potential cycling in PEMFC. Different potential profiles between 0.60 and 1.0 V vs. RHE were investigated: a 3 s – 3 s square wave (inspired by the standardised AST protocol suggested by the Fuel Cell Technical Team of the U.S Department of Energy, DoE/FCTT^22^) and triangular waves at different potential sweep rates (5, 10, 100, 500 and 1000 mV s^−^^1^), the latter being often found in the literature (see associated voltammograms in Supplementary Figs. S13, S14). The results presented in Fig. 5 unambiguously reveal a pronounced effect of the applied potential profile and sweep rate on Pt structural response in PEMFC. Quantifying oxygen surface coverage from lattice constant variation in Fig. 5a, b, whereas maximum oxygen surface coverage reached at 1.0 V vs. RHE is nearly equivalent between the square wave and the 5 mV s^−1^ triangular wave, the maximum oxygen coverage sharply decreases for triangular waves performed at higher sweep rates. Also, the minimum oxygen surface coverage at 0.60 V vs. RHE remains higher for any of the triangular profiles compared to the square wave, with a clear dependence on the sweep rate. The overall Kernel density estimations of the lattice constant values for the different profiles in Fig. 5c make explicit the transition from distinct reduced-oxidised bimodal behaviour toward a partially reduced unimodal state as the sweep rate increases. This naturally translates into the extent of maximum surface crystallinity losses associated with the metal-oxide phase transition in Fig. 5d. Clearly (and as quantified as ‘cycle depth’ in Fig. 5e), the amount of extracted Pt during a cycle significantly drops for any of the triangular profile applied compared to the square wave, with a clear dependence on the sweep rate. In the frame of quantifying the degradation power of an AST profile, one can define the ‘cycle efficiency’ being the cycle depth normalised by the cycle time period. As shown in Fig. 5f, the cycle efficiency of the square-wave profile is near 35-, 24-, 10- and >100-fold higher than the triangular waves at 5, 10, 100, and > 500 mV s^−1^, respectively.

These observations largely agree with previous conclusions^46^ and support the choice of the Fuel Cell Technical Team of the U.S. Department of Energy (DoE/FCTT) in suggesting a square-wave profile as a standardised AST protocol. However, it is clear that only 3 s of polarisation at 1.0 V vs. RHE is insufficient to reach the steady state of oxidation. In light of the time constants reported here, 10 s polarisation is required to induce the Pt oxidation to be encountered in a PEMFC device. The faster reduction process, however, is largely completed in 3 s, which is consistent with the time constant reported (ca. 310 ms). A more rational AST protocol between 0.6 and 1.0 V vs. RHE would thus comprise a 3 s – 10 s square wave profile. Finally, the above conclusions reached from ASTs conducted in PEMFC mostly transpose to model experiments on thin-films at room temperature. Because the time constant of the slowest step in oxidation is found to be 1.15 s, 95 % of the oxidation proceeds in 3.45 s, and the steady state is not reached during the 3 s – 3 s square wave (see Supplementary Fig. S15). In that case, a 3 s – 4 s square wave profile would be more suited, although the practical irrelevance of ASTs conducted at room temperature is here further demonstrated^25,47^.

In summary, ultra-fast operando WAXS in both PEMFC and model electrochemical environment has been successfully employed to investigate Pt oxidation in PEMFC. The major finding is that the onset potential for metal-oxide phase transition, i.e., the extraction of Pt surface atoms from their metallic crystal phase lattice position, in PEMFC, is as low as 0.80 V vs. RHE, which is far below the commonly accepted value and falls right at the centre of PEMFC cathode potential range of operation. Such early Pt oxidation in PEMFC has consequences for its ORR activity, as the PEMFC performance was found to correlate to Pt surface oxidation recovery during cathodic polarisation under conditions minimising O_2_ mass-transport limitation. Beyond affecting Pt activity, electrochemical on-line ICP-MS experiments performed above ambient temperature and at low pH suggest such early extracted Pt is particularly prone to anodic transient dissolution at the OCP (20.5-fold increase compared to model conditions at 1.0 V vs. RHE while only 8.3-fold at 1.23 V vs. RHE). Finally, the determination of first-order time constants associated with the metal-oxide phase transition revealed an overall slow process, which requires more than 10 s for near-complete oxidation at 1.0 V vs. RHE. This defines fundamental requirements for rational AST protocols, for which informed potential cycle and profile must be used. At a fixed potential window (here 0.6 and 1.0 V vs. RHE), the presented data suggest abandoning triangular waves of any potential sweep rate to the benefit of 3 s – 10 s square waves.

Overall, efficient Pt oxidation mitigation strategies in PEMFC must consequently be found to significantly improve PEMFC performance and durability. From the material science perspective, disabling Pt oxidation seems an arduous, but not impossible task as atomic decoration^48^ or particle encapsulation strategies have already shown promising results^49,50^. Complementarily, PEMFC system management strategies in the fashion of those currently employed to protect the cathode from high potential excursions during reactant gas starvation or start-up/shut-down events could be adapted to potential limits even below the OCP.

## Methods

### Materials

Commercial membrane electrode assemblies (MEAs) were purchased from the Fuel Cell Store and used in PEMFC without further treatment. The 5-layer, 4.84 cm^2^, MEAs were composed of a 60 wt.% carbon-supported Pt/C catalyst loaded at 0.5 mg_Pt_ cm^−2^ for both anode and cathode, a 50.8 µm thick Nafion® NRE-212 membrane, and woven carbon cloth with microporous layer as gas diffusion layers. A 40 wt.% carbon-supported Pt/C was purchased from Johnson Matthey (JM) and used in thin-film electrochemical flow cell without any further treatment.

### X-ray transparent fuel cell operation

The MEA was mounted in a house-made X-ray transparent fuel cell largely described in previous contributions^33–36^. The cell was operated at 80 °C with H_2_/O_2_ or H_2_/N_2_ at anode/cathode, 65 °C dewpoint (52% relative humidity) and 100 mbar backpressure. After a break-in procedure consisting of a 1 h voltage hold at 0.60 V in H_2_/O_2,_ the cathode was purged with N_2_ to both fix and minimise ohmic voltage losses for the cyclic voltammetry experiments. The cell voltage was controlled by a SP-240 potentiostat (Biologic) equipped with a 4 A booster. Prior to any cyclic voltammetry experiment, the high-frequency resistance (typically 0.6 Ohm at 200 kHz) was measured by means of potentio electrochemical impedance spectroscopy (PEIS), and the potential of the working electrode was dynamically compensated for 85% of the ohmic losses. Under these conditions of cell temperature, low current density and gas backpressure, the anode can be reasonably associated to an RHE (< 2 mV shift), and the cathode potential vs. RHE given by the cell voltage.

### Conventional fuel cell operation

The MEA was mounted in a commercial single-cell PEMFC casing (Scribner) and operated at 80 °C with H_2_/Air or H_2_/O_2_ at anode/cathode, 80 °C dewpoint (100% relative humidity) and 500 mbar backpressure with a house-made test bench. The cell voltage was controlled by a SP-150 potentiostat (Biologic) equipped with a 20 A booster. The break-in procedure was conducted in H_2_/Air (313/496 sccm) and consisted of potential cycles between the open circuit voltage (OCV, 10 s), a 100 mV s^−1^ potential sweep from the OCV to 0.10 V, followed by 1 min hold at 0.10 V. The cycles were repeated for a total duration of 1 h. The oxidant gas was then turned to O_2_, and the polarisation curve was recorded using the staircase potentio electrochemical impedance spectroscopy with a total of 14 potential steps (3 min hold) between the OCV and 0.10 V. The high-frequency resistances measured at the end of all steps were used to later correct the potential values from the ohmic losses (100%).

### X-ray transparent thin-film electrochemical flow cell operation

10 µL of an ink suspension composed of 5 mg of 40 wt.% Pt/C catalyst (JM), 3600 µL of 18.2 MΩ ultra-pure water (Millipore), 1446 µL of isopropanol and 20 µL of 5 wt.% Nafion solution (Electrochem. Inc.) was deposited on a 0.196 cm^2^ glassy carbon substrate, thus reaching a Pt loading of 20 ${{{\rm{\mu }}}{{\rm{g}}}}_{{{\rm{Pt}}}}\,{{{\rm{cm}}}}^{-2}$. The sample was mounted in a 3-electrode flow cell^30,31^. A Pt wire (2 mm diameter and 2 cm long) was used as a counter electrode, and a commercial Ag/AgCl electrode (ET072, eDAQ) as the reference electrode. The ~ 50 mL cell was fed with 0.10 M HClO_4_, (Suprapur®, Merck) at a rate of 20 mL min^−1^ using a peristaltic pump from a 500 mL reservoir. The electrochemistry was controlled by a SP-300 potentiostat (Biologic). The catalyst was first conditioned using 50 cycles between 0.05 and 1.23 V vs. RHE at 500 mV s^−1^. The temperature of the room was regulated at 25 °C. The cell high-frequency resistance (typically 27 Ω in 0.1 M HClO_4_) was used to correct the electrode potential for 85 % of the ohmic drop in a dynamic fashion.

### Stroboscopic *operando* synchrotron wide-angle X-ray scattering (WAXS) measurements

Synchrotron WAXS measurements were performed at the ID31 beamline of the European Synchrotron Radiation Facility (ESRF) in Grenoble, France. The high energy X-ray beam (77 keV) was focused on the sample through the cells in grazing incidence configuration, and the scattered signal was collected using a Dectris Pilatus CdTe 2 M detector. The energy, detector distance and tilts were calibrated using a standard CeO_2_ powder, and the 2D diffraction patterns were reduced to the presented 1D curves using the pyFAI software package^51^. For a fair comparison between cyclic voltammetry experiments recorded at different potential sweep rates, the chosen temporal resolution for the reconstructed WAXS patterns was kept constant at ca. 1/60^th^ of the cycle period *T*. The WAXS measurements were performed on stable cyclic voltammograms.

### Rietveld refinement of the WAXS patterns

Rietveld refinement of the WAXS patterns was performed to extract the phase structure, crystallite size and lattice parameter using the *Fm3m* structure of Pt and the Fullprof software. The instrumental resolution function was determined by the refinement of a CeO_2_ standard sample. Thomson-Cox-Hastings profile function was adopted^52^. The background of patterns was described by an interpolated set of points with refinable intensities.

### Pair distribution function (PDF) analysis

PDFs were obtained from the WAXS patterns using the PDFGetX3 software^53^ up to *Q*_max_ = 11.7 Å^−1^, and the instrumental resolution parameters were determined from the refinement of a standard CeO_2_ sample. The PDFs were fitted using the fcc structure of Pt with the PDFGUI software^54^, and the refined parameters where the phase scale factor, the unit cell lattice constant, delta2 (used to account for correlated atomic motion effects), the isotropic coherent structural domain size, and an overall a.d.p.

### First-order rate constant determination

The lattice constant and scattered intensity variations during potential steps were fitted using a linear combination of exponential functions of the type:

1 $$y={y}_{0}+{A}_{1}{e}^{-(x-{x}_{0})/{t}_{1}}+{A}_{2}{e}^{-(x-{x}_{0})/{t}_{2}}$$

where ${y}_{0}$ and ${x}_{0}$ are the fitted boundary conditions, ${A}_{1}$ and$\,{A}_{2}$ the fitted amplitude variations, and ${t}_{1}$ and ${t}_{2}$ are the fitted first-order time constants. The two exponential functions were found necessary to capture the oxidation process while only one was sufficient to capture the reduction. More details and numerical values of the fitted parameters can be found in Supplementary Fig. S11 and Supplementary Table S1.

### Electrochemical on-line inductively coupled plasma mass spectrometry

A commercial electrochemical flow cell (BASi, MF-1092, cross-flow cell) was used. The flow cell consists of two glassy carbon disks (3 mm) aligned in series, which are embedded into PEEK material. The first disk was used as the counter electrode, and the working electrode was in the direction of the electrolyte flow. 3.4 µL of an ink suspension composed of 5.4 mg of 40 wt.% Pt/C catalyst (JM), 1800 µL of 18.2 MΩ ultra-pure water (Millipore), 723 µL of isopropanol and 29 µL of 5 wt.% Nafion solution (Electrochem. Inc.) was deposited on the 0.071 cm^2^ glassy carbon substrate thus reaching a Pt loading of 20 ${{{\rm{\mu }}}{{\rm{g}}}}_{{{\rm{Pt}}}}\,{{{\rm{cm}}}}^{-2}$. An Ag/AgCl reference electrode was used. The ~ 4 µL cell was fed with an aqueous electrolyte at a rate of 400 µL min^−1^ using a peristaltic pump. The electrochemistry was controlled by an SP-300 potentiostat (Biologic). The electrolyte was either 0.10 M HClO_4_ (99.999 % trace metal basis, Merck) at room temperature (26 °C), 1.0 M HClO_4_ at room temperature (28 °C) or 1.0 M HClO_4_ at 60 °C (temperature at the cell outlet). In this last case, the electrolyte was heated prior to entering the electrochemical cell (see Supplementary Information). The potential shift of the Ag/AgCl reference electrode with temperature was calibrated in situ by measuring the open circuit voltage of the Pt/C working electrode after hydrogen evolution in the cell at both temperatures (considering the Pt/C catalyst under locally saturated HClO_4_ electrolyte a reversible hydrogen electrode). The flow cell was connected to an Agilent 7900 ICP-MS equipped with a Micromist/Scott nebuliser. The ^195^Pt metal ions were detected with 0.1 s integration time per point. Before each measurement, calibration curves were carried out using daily prepared standard solutions of ^195^Pt (0, 10, 100, 1000 and 10000 ppt). The catalyst was conditioned also using 50 cycles between 0.05 and 1.23 V vs. RHE at 500 mV s^−1^. The associated Pt electrochemical surface area used for the normalisation of the ICP-MS data was measured separately using the CO_ads_ stripping technique.

### Computational details

Periodic DFT calculations were performed using the Vienna Ab Initio Simulation Package (VASP)^55,56^ within the generalised gradient approximation (GGA) using PBE^57^ functional for exchange and correlation potential and projector augmented wave pseudopotentials (PAW)^58^ with a cut-off energy of 450 eV. 405 Pt atoms truncated-octahedral clusters were computed with up to 240 oxygen atoms adsorbed on their facets. O atoms were adsorbed on the most stable fcc sites of the {111} facets and on the {100} ones converging into a threefold-like site. They were set in a 3 x 3 x 3 nm^3^ cubic unit cell. The intermolecular facet distances between periodic surfaces were larger than 9 Å. Gamma point calculations were used, and structural relaxations were performed on all atoms. The residual forces after structural relaxation were lower than 0.01 eV/Å. Simulated diffraction patterns were obtained from the atomic positions and the Debye formula^59^, as further detailed in the Supplementary Information.

### Transmission electron microscopy

Transmission electron microscopy images of the investigated catalysts were obtained using a Jeol 1400 Flash microscope operated at 120 kV with a point-to-point resolution of 0.38 nm. The images were used to build the particle size distribution of the catalysts by measuring the apparent diameter of > 500 nanoparticles per sample.

## Supplementary information

Supplementary Information

Transparent Peer Review file

## Source data

Source Data

## Data Availability

All data supporting the findings in this study are available within the paper and the Supplementary Information/Source Data file. Main operando WAXS data recorded during cyclic voltammetry in PEMFC and computed Pt-405 nanoparticles models generated in this study have been deposited in the figshare database under accession code 27902325. Source data are provided in this paper.
