# Supplementary material for: Metal-oxide phase transition of platinum nanocatalyst below fuel cell open-circuit voltage
Source: Nat Commun. 2025 Jan 22;16:936. doi: 10.1038/s41467-024-55299-3 (PMC11754633; doi:10.1038/s41467-024-55299-3)
Supplement: Supplementary file 1 — Supplementary Information [file 41467_2024_55299_MOESM1_ESM.pdf]

Supplementary Information

Metal-oxide phase transition of platinum nanocatalyst below fuel cell open-circuit voltage

*Carlos A. Campos-Roldán [1], Amir Gasmi [1], Meryem Ennaji [1], Morgane Stodel [2], Isaac Martens [3], Jean-Sébastien Filhol [1], Pierre-Yves Blanchard [1], Sara Cavaliere [1], Deborah Jones [1], Jakub Drnec [3] and Raphaël Chattot [1, *]*

[1] ICGM, Univ. Montpellier, CNRS, ENSCM, 34095 Montpellier Cedex 5, France

[2] CIRIMAT, Université Toulouse 3 Paul Sabatier, Toulouse INP, CNRS, Université de Toulouse, 118 Route de Narbonne, 31062 Toulouse cedex 9 - France

[3] ESRF, The European Synchrotron Radiation Facility, 71 Avenue des Martyrs, CS40220, 38043 Grenoble Cedex 9, France

*e-mail: (RC) raphael.chattot@umontpellier.fr

SUPPLEMENTARY METHODS

Estimation of the 'Surface Atoms Ratio' *D*. This parameter was estimated using the mathematical model of face-centred cubic cuboctahedral particles from Montejano-Carrizales *et al.* [9,10]:

$$D = 100\frac{Ns}{N} \quad \text{Eq. S1}$$

where *N* and *N*s are the total number of atoms and the number of surface atoms, respectively:

$$N = 10\frac{m^3}{3} + 5m^2 + \frac{11}{3}m + 1 \quad \text{Eq. S2}$$

$$Ns = 10m^2 + 2 \quad \text{Eq. S3}$$

'*m*' corresponds to the number of atomic layers composing the particle. The values of '*m*' are linked to the particle size '*d*' by the relation:

$$m = \frac{d}{2\sqrt{3}r_{\mathrm{Pt}}} \quad \text{Eq. S4}$$

With 'r_{Pt}' the covalent radius of a Pt atom (r_{Pt}= 0.135 nm).

In this study, *D* is calculated from the average particles' size observed by TEM.

Computational details. Periodic DFT calculations were performed using the Vienna *Ab Initio* Simulation Package (VASP) [13,14] within the generalized gradient approximation (GGA) using PBE [15] functional for exchange and correlation potential and projector augmented wave pseudopotentials (PAW) [16] with a cut-off energy of 450 eV. 405 Pt atoms truncated-octahedral clusters were computed with up to 240 oxygen atoms adsorbed on their facets. O atoms were adsorbed on the most stable *fcc* sites of the {111} facets and

on the {100} ones converging into a threefold-like site. They were set in 3x3x3 nm^3 cubic unit cell. The intermolecular facet distances between periodic surfaces were larger than 9 Å. Gamma point calculations were used and structural relaxations were performed on all atoms. The residual forces after structural relaxation were lower than 0.01 eV/Å.

Computed diffraction patterns were obtained from the atomic positions and the Debye formula [17]:

$$I_{Debye}\left(\frac{Q}{2\pi}\right) = \sum_i \sum_j f_i(Q) f_j(Q) \frac{\sin(Qr_{ij})}{Qr_{ij}} \quad \text{(eq. S6)}$$

Where:

- $Q = {}^{4\pi}/_{\lambda} \sin(\theta)$ is the momentum transfer, λ is the wavelength and θ is diffraction half-angle,
- r_{ij} is the distance between atoms *i* and *j*,
- $f_i(Q)$ is the atomic form factor of atom *i* as a function of Q.

$f_i(Q)$ for Pt and O atoms were approximated using [18]:

$$f(Q) = \sum_{i=1}^{4} a_i e^{-b_i(\frac{Q}{4\pi})^2} + c \quad \text{(eq. S7)}$$

Where the coefficients for Pt and O atoms are:

Element	a_1	b_1	a_2	b_2	a_3	b_3	a_4	b_4	c
**Pt**	27.0059	1.51293	17.7639	8.81174	15.7131	0.42493	5.7837	38.6103	11.6883
**O**	3.0485	13.2771	2.2868	5.7011	1.5463	0.3239	0.867	32.9089	0.2508

The as-obtained computed diffraction patterns I_{Debye} (*Q*) where then compared to the experimental ones *I*(*Q*) in **Figure S6.a-b** using a least-square minimization of the type:

$$\boldsymbol{S} = \sum_i (\boldsymbol{I}(\boldsymbol{Q}_i) - (\boldsymbol{A} \cdot \boldsymbol{I}_{Debye}\left(\frac{\boldsymbol{k}+100}{100} \cdot \boldsymbol{Q}_i\right) + \boldsymbol{B}))^2 \quad \text{(eq. S8)}$$

Where:

- *A* is the scale factor,

- B is a constant background value,

k is a parameter (in %) allowing quantifying mismatch between computed and experimental Q values possibly due to theoretical simplifying hypothesis or experimental errors.

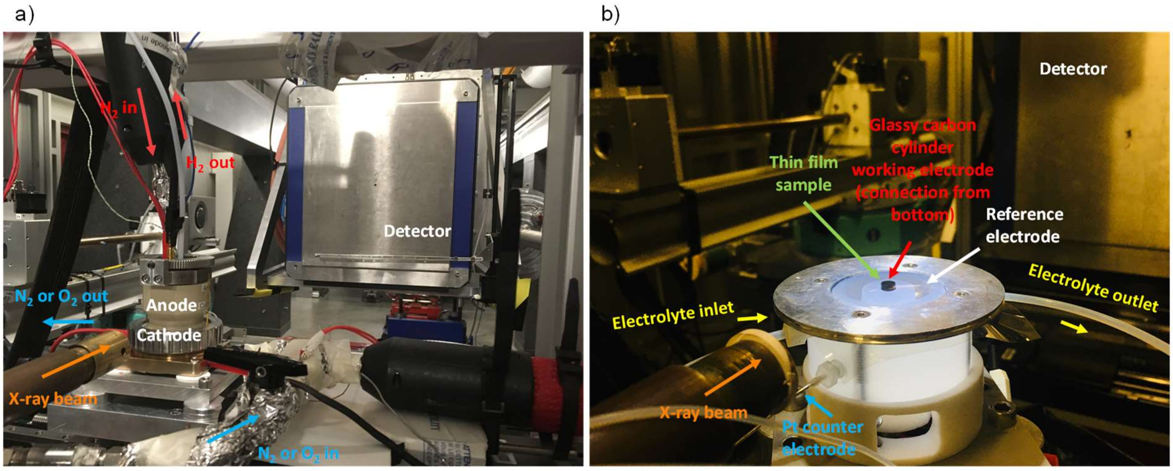

Figure S1: Setups for *operando* high energy X-ray scattering experiments. a) X-ray transparent PEMFC and b) thin-film electrochemical flow cell.

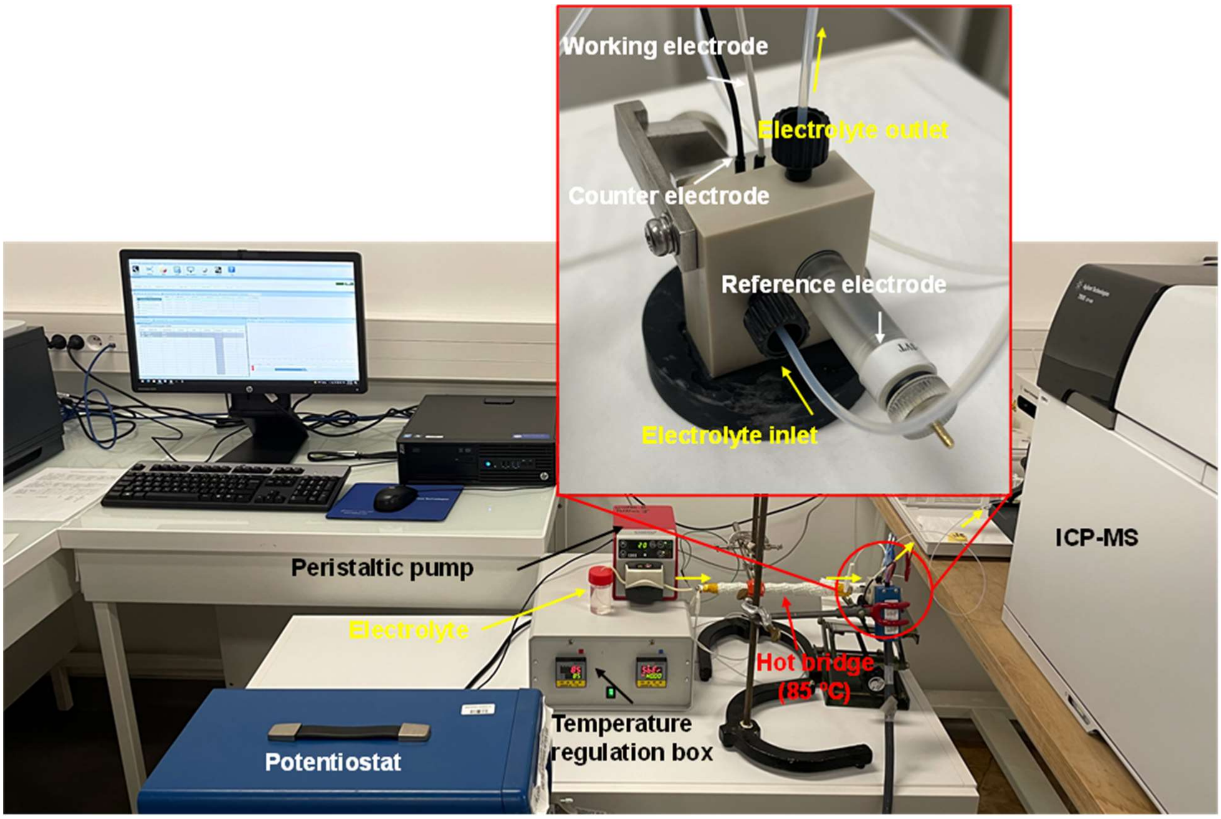

Figure S2: Setup for the electrochemical on-line ICP-MS experiment at controlled temperature

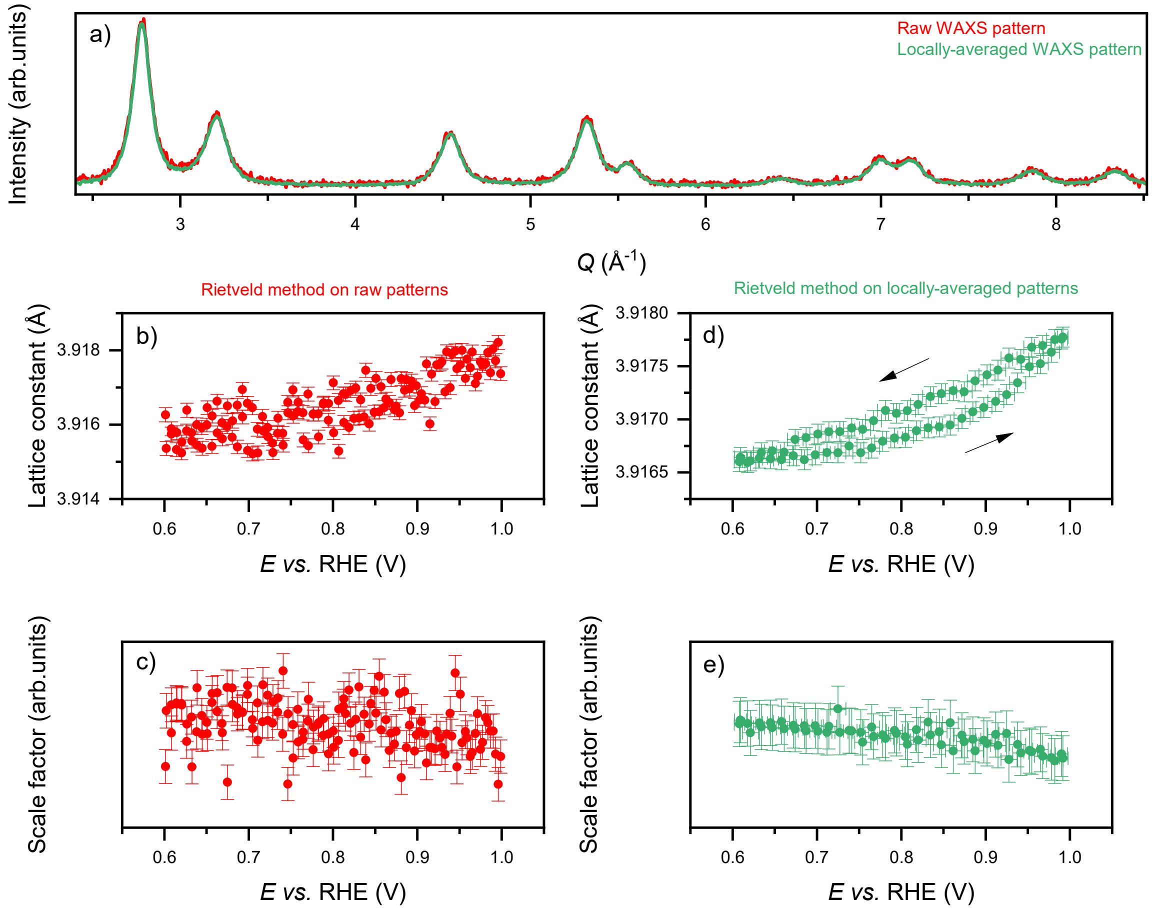

Figure S3: Effect of the used WAXS data processing on the refinement of Pt/C structural parameters during cyclic voltammetry at 1000 mV s^{-1}. a) example of raw *vs.* locally-averaged WAXS patterns, refined b) lattice constant and c) scale factor from raw patterns, refined d) lattice constant and e) scale factor from locally-averaged patterns using strictly similar refinement model. Errors bars correspond to the standard deviations associated with the refinement of the different parameters.

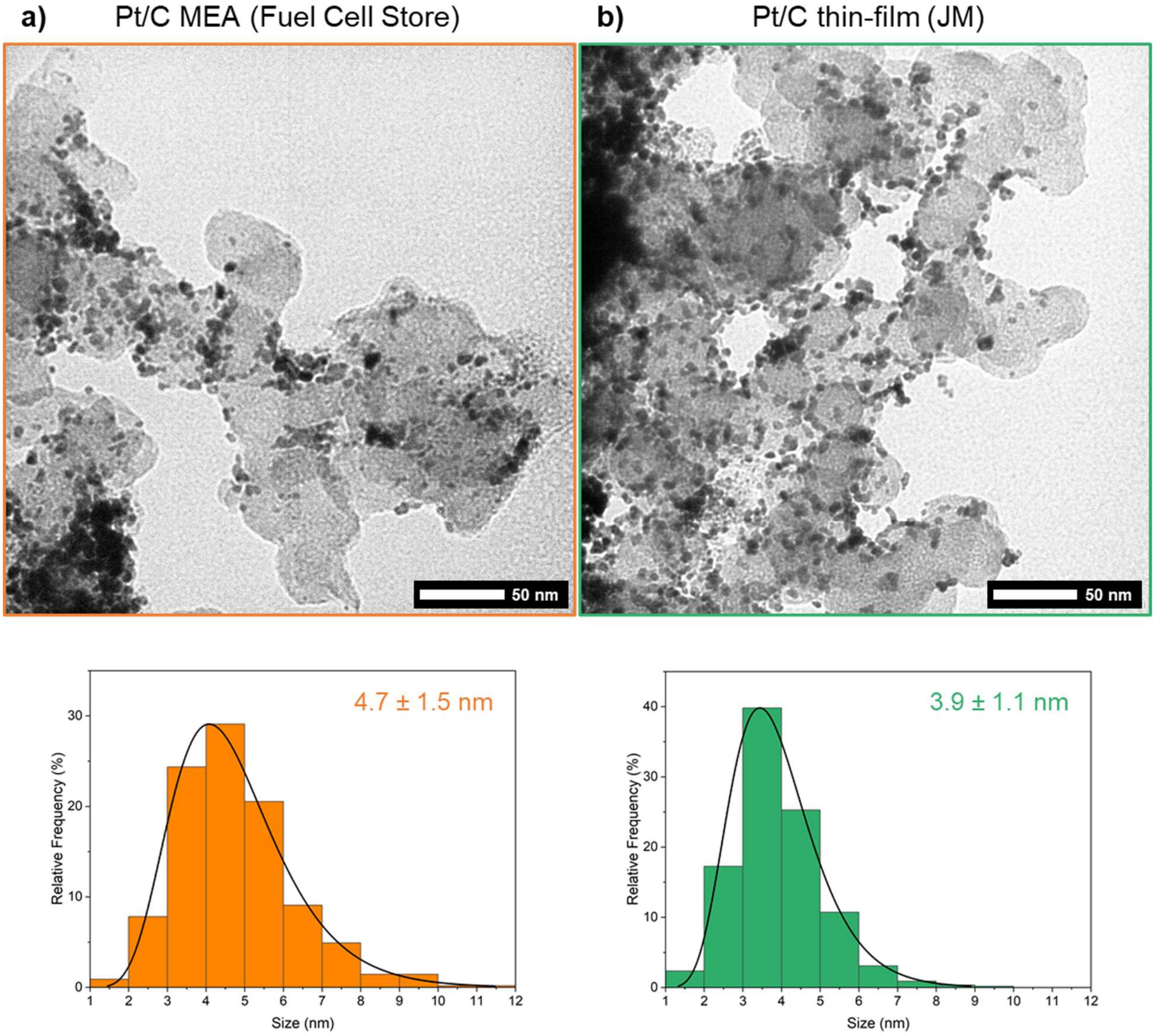

Figure S4: Representative transmission electron microscopy images and associated particle size distribution of a) the Pt/C cathode catalyst used in PEMFC and b) the Pt/C catalyst powder used in thin-film configuration. The images were used to build the particle size distribution of the catalysts from measuring the apparent diameter of > 500 nanoparticles per sample.

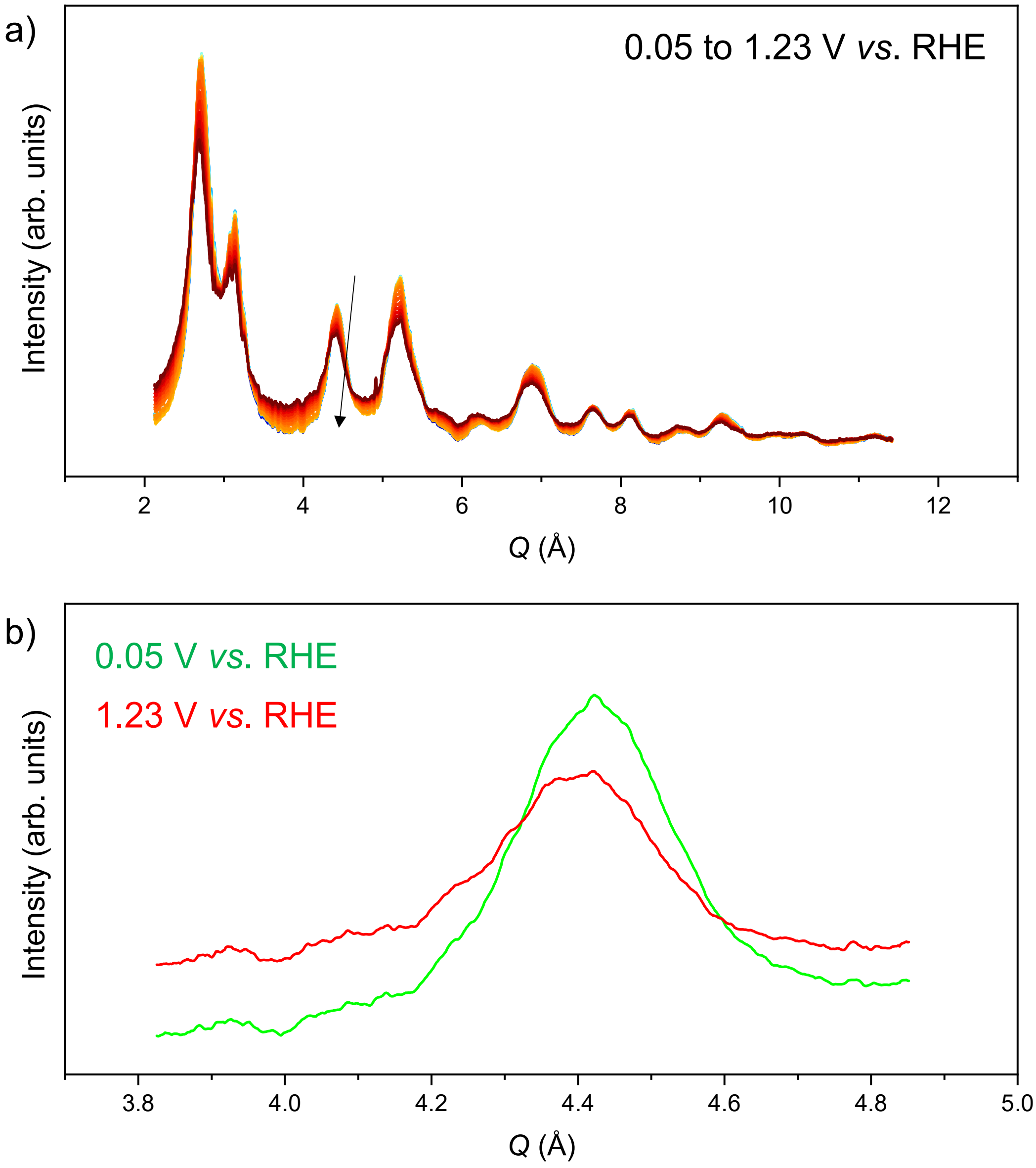

Figure S5: Changes in experimental WAXS patterns observed during Pt oxidation. a) WAXS patterns recorded during the reconstructed cyclic voltammetry experiment in PEMFC between 0.05 and 1.23 V vs. RHE at 20 mV s-1 (anodic scan only). A fixed background measured prior to oxidation was removed; b) focus on [220] reflection before and during oxidation.

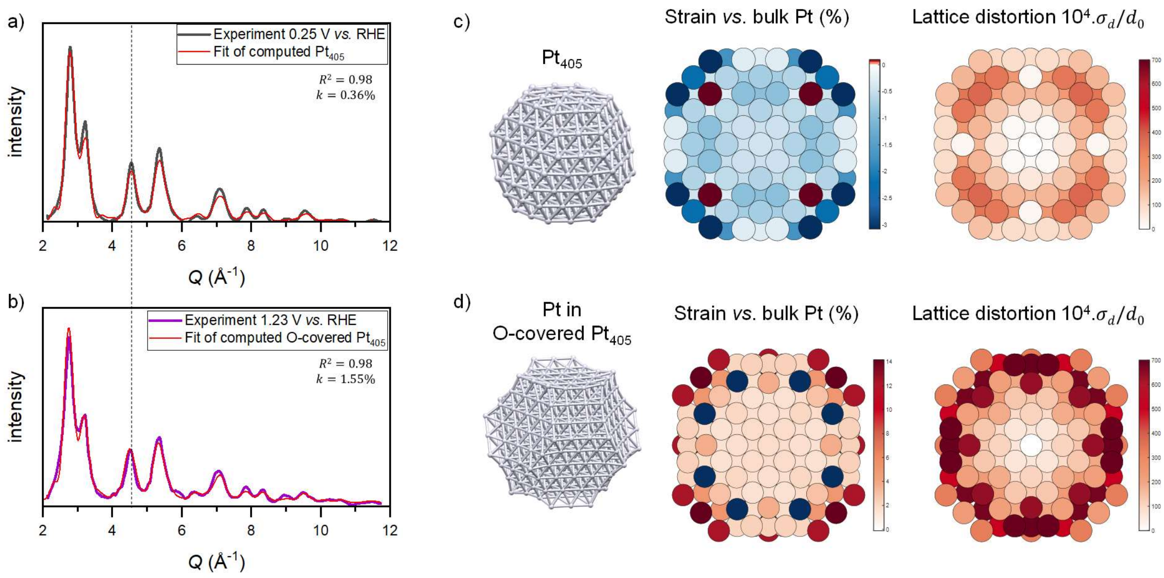

Figure S6: Computed 405-atom Pt nanoparticles covered with adsorbed hydrogen or oxygen. Fits of computed patterns on experimental patterns for a) Pt_{405} without adsorbate and b) O-covered Pt_{405}; c) visual representation of Pt atoms only, strain and lattice distortion maps for c) Pt_{405} without adsorbate and d) O-covered Pt_{405}. In a)-b), the computed XRD patterns were obtained *via* the Debye formula applied to the optimized structures. In c)-d) the maps where obtained by analysing the interatomic distances of each Pt atom and its first coordination shell. The strain is derived from the mean distance and the lattice distortion by its standard deviation.

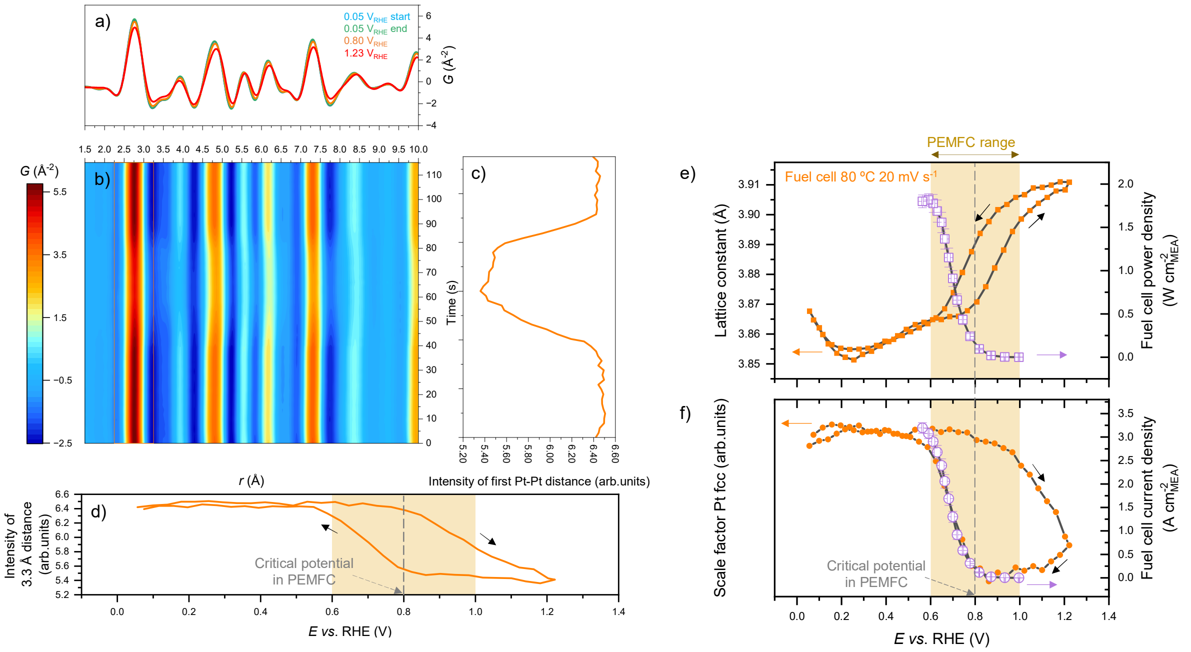

Figure S7: Pair distribution function (PDF) analysis of the *operando* WAXS patterns collected during cyclic voltammetry experiment between 0.05 and 1.23 V *vs.* RHE at 20 mV s^{-1} in PEMFC. a) Selected PDF curves at different electrode potentials, b) colormap showing the PDF signals as a function time, c) integrated intensity of the peak at $r = 2.77 \pm 0.5$ Å (red box in b)) corresponding to the first Pt-Pt distance, d) integrated intensity of the first Pt-Pt distance as a function of the electrode potential, e) evolution of the lattice constant obtained by fitting the PDF data with a Pt fcc phase as a function of the electrode potential and f) evolution of the scale factor of the fitted Pt fcc phase as a function of the electrode potential. The fuel cell for operando WAXS was operated at 80 °C, H_2/N_2 at anode/cathode, 65 °C dewpoint, 100 mbar backpressure, and the electrochemical flow-cell at controlled room temperature of 25 °C. The fuel cell power density (purple square) and current density (purple dots) plotted in e) and f), respectively, were measured in a separated dedicated experiment on similar MEA and the cell was operated at 80 °C, H_2/O_2, 313/496 sccm at anode/cathode, 80 °C dewpoint, 500 mbar backpressure after 3 min of stabilization per point. The cell voltage was corrected at each point by the cell high frequency resistance. The orange boxes in d)-f) represent the PEMFC practical voltage range between 0.60 and 1.0 V vs. RHE, corresponding to the voltage of maximum power density and the open circuit voltage, respectively, as shown in e). Errors bars correspond to the standard deviations associated with the refinement of the different parameters

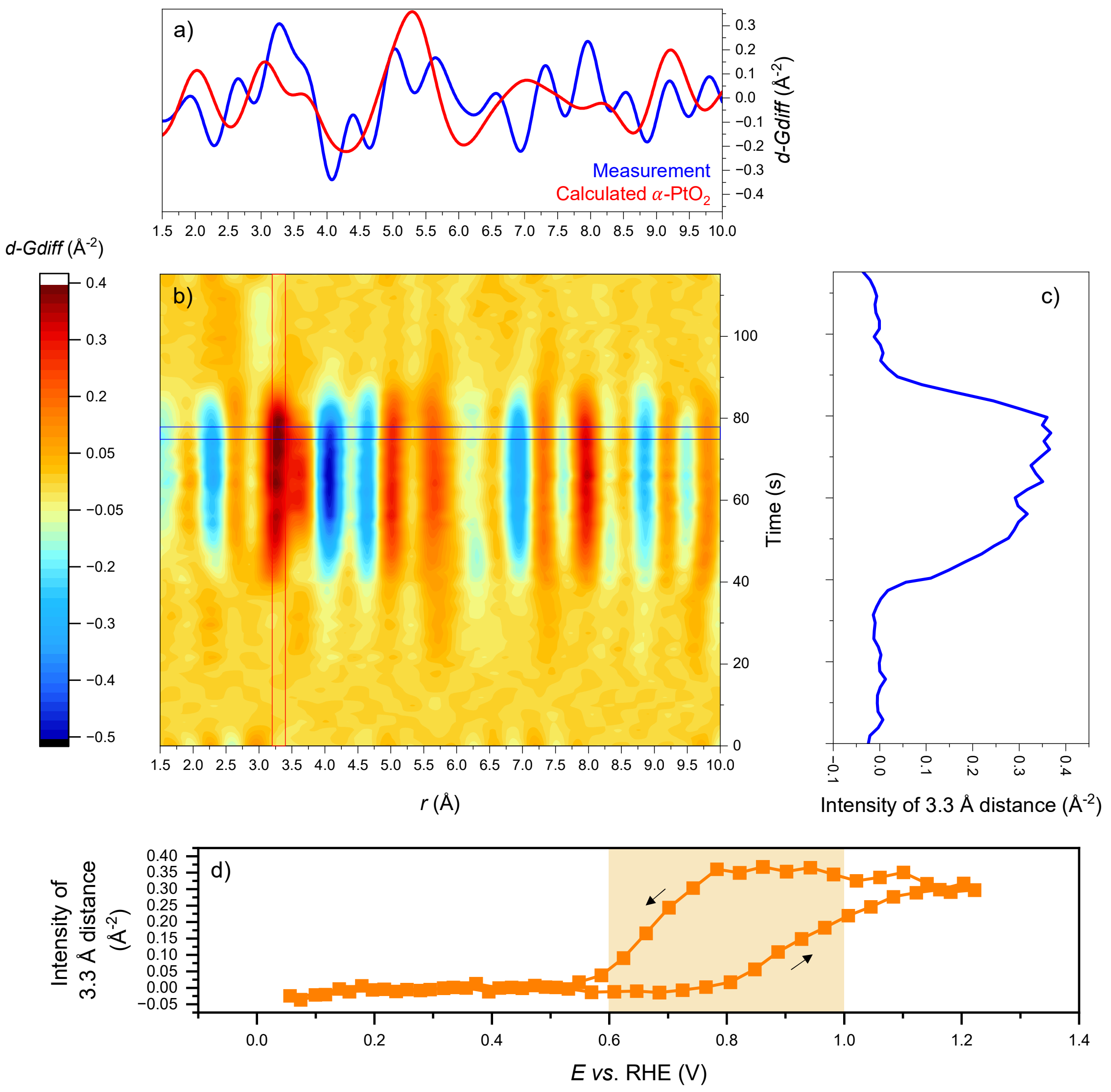

Figure S8: Emergence of non-face-centered-cubic (fcc) Pt-Pt interatomic distances from analysing the residuals from the PDF fits with an fcc Pt unit cell. Differential PDFs residuals *d-Gdiff* obtained after fitting the PDFs with a Pt fcc unit cell during cyclic voltammetry experiment between 0.05 and 1.23 V vs. RHE at 20 mV s^{-1} in PEMFC. The fit residual *Gdiff* obtained from reduced Pt electrode was subtracted to all other *Gdiff* curves to obtain the differential *d-Gdiff* and cancel out all systematic errors. a) *d-Gdiff* curve at the maximum signal intensity (blue) and calculated PDF signal of α-PtO_2 unit cell; b) colormap showing the *d-Gdiff* signals as a function time, c) mean intensity of the peak at $r = 3.3 \pm 0.2$ Å (red box in b)), d) integrated intensity of the $r = 3.3 \pm 0.2$ Å distance as a function of the electrode potential. The Figure shows the growing oxide does not necessarily matches the local structure of α-PtO_2.

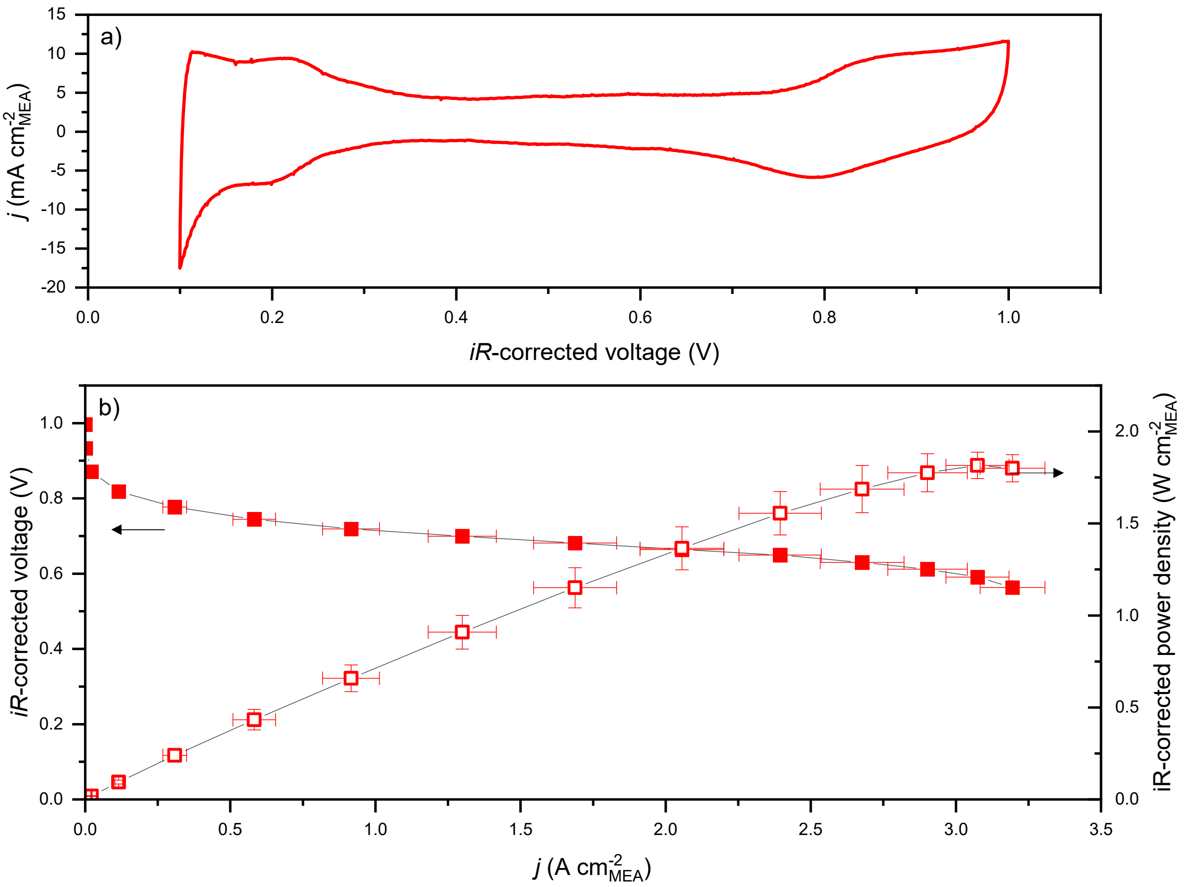

Figure S9: MEA performance in PEMFC. a) cyclic voltammogram recorded at 50 mV s^{-1} in H_2/N_2. b) current density (left axis, solid markers) and power density (right axis, hollow markers) recorded during polarization curve in H_2/O_2 after 3 min stabilization per point. The cell operated at 80 °C with 313/496 sccm at anode/cathode, 80 °C dewpoint (100 % relative humidity) and 500 mbar backpressure with a house-made test bench. The cell voltage is corrected from the high frequency resistance measured at each point. In b), the markers and error bars correspond to the average and standard deviation values, respectively, of two independent measurements.

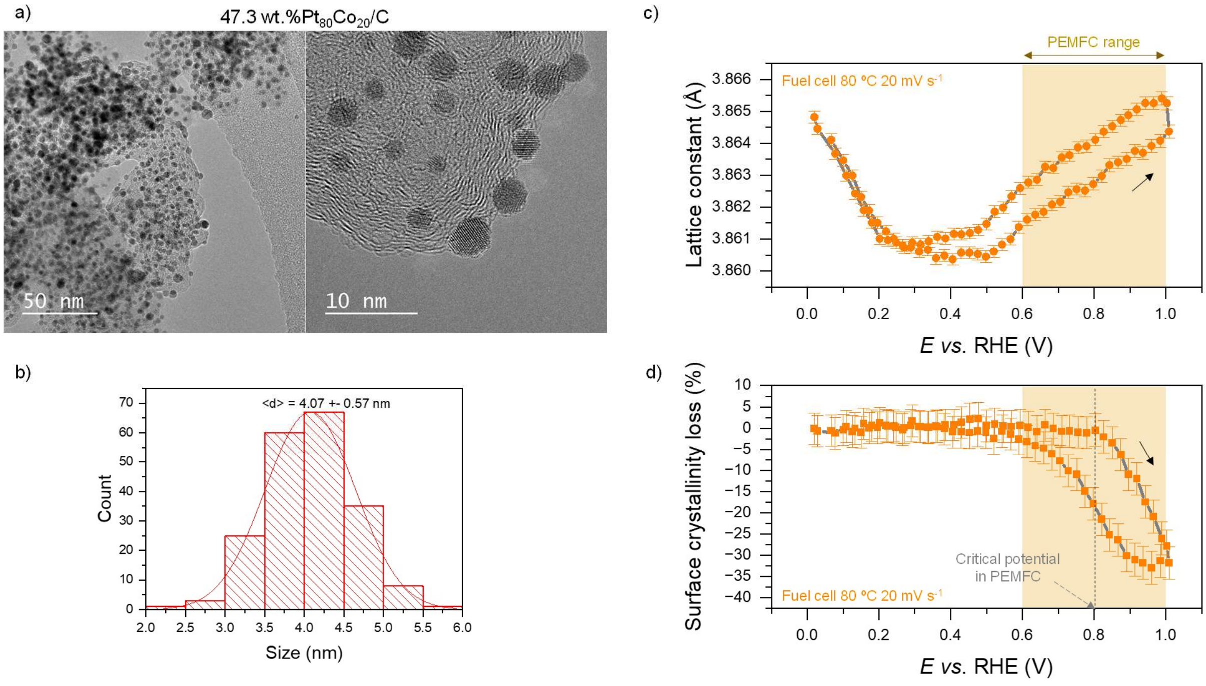

Figure S10: Generalization of the results to a PtCo/C catalyst. a) HRTEM images of the 47.3 wt.% $Pt_{80}Co_{20}$/C catalyst investigated (more details available on request); b) associated particles' size distribution; c) Pt lattice constant variations and b) surface-fraction-normalized scattering intensity variations during cyclic voltammetry experiment in PEMFC between 0.05 and 1.0 V vs. RHE at 20 mV s^{-1}. Errors bars correspond to the standard deviations associated with the refinement of the different parameters.

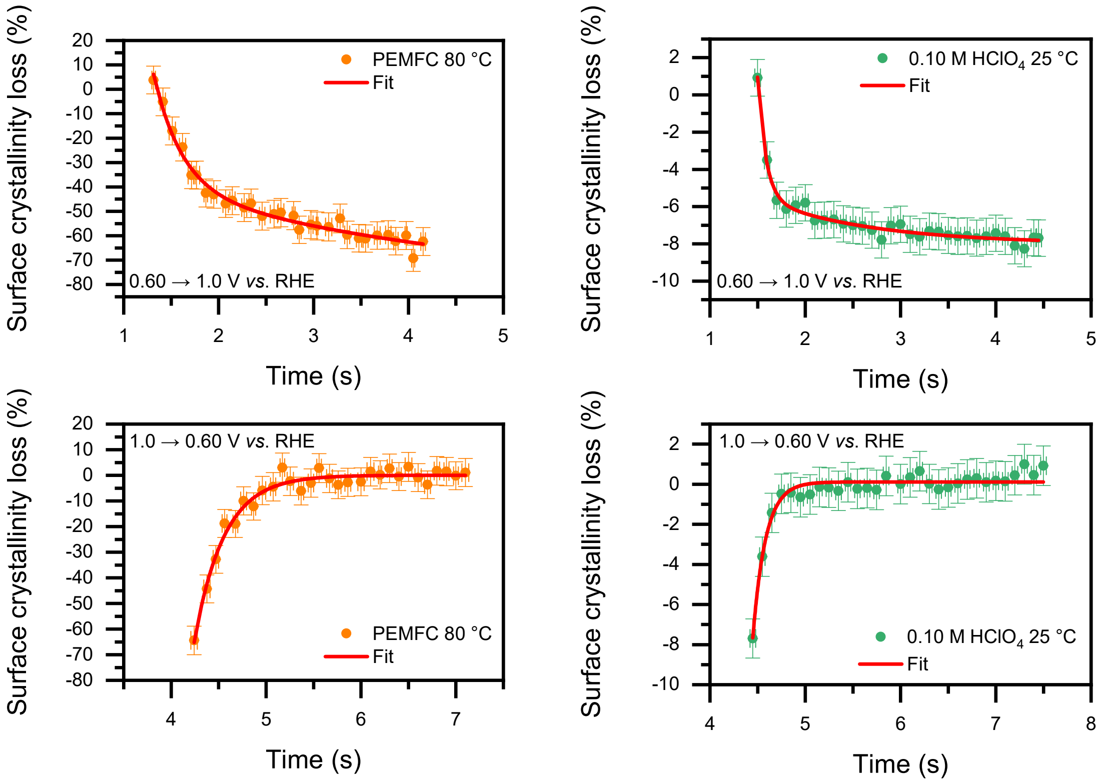

Figure S11: Fits associated with the surface crystallinity loss or recovery during oxidation and reduction in both PEMFC and thin-film electrochemical cells. Errors bars correspond to the standard deviations associated with the refinement of the different parameters.

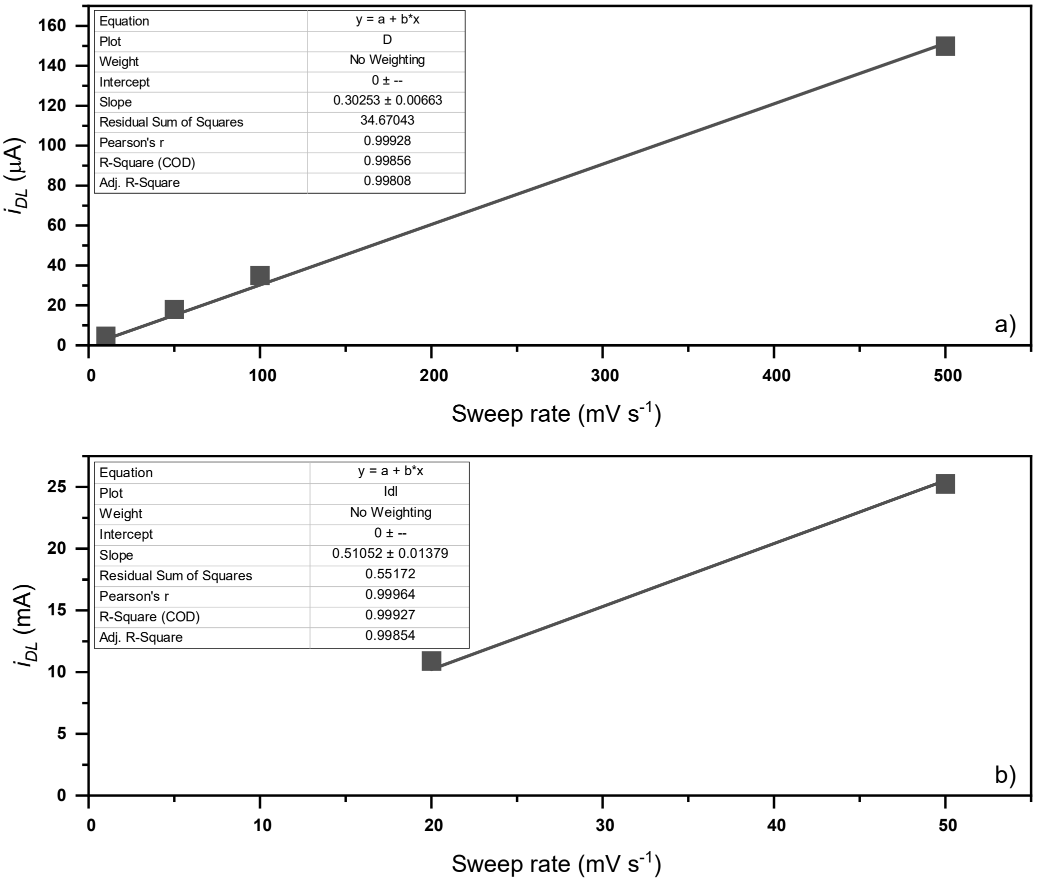

Figure S12: Estimation of the double-layer capacitance C_{DL} from linear regression of double-layer currents measured at $E = 0.40$ V *vs*. RHE at different potential sweep rates for a) the thin-film flow cell and b) the PEMFC.

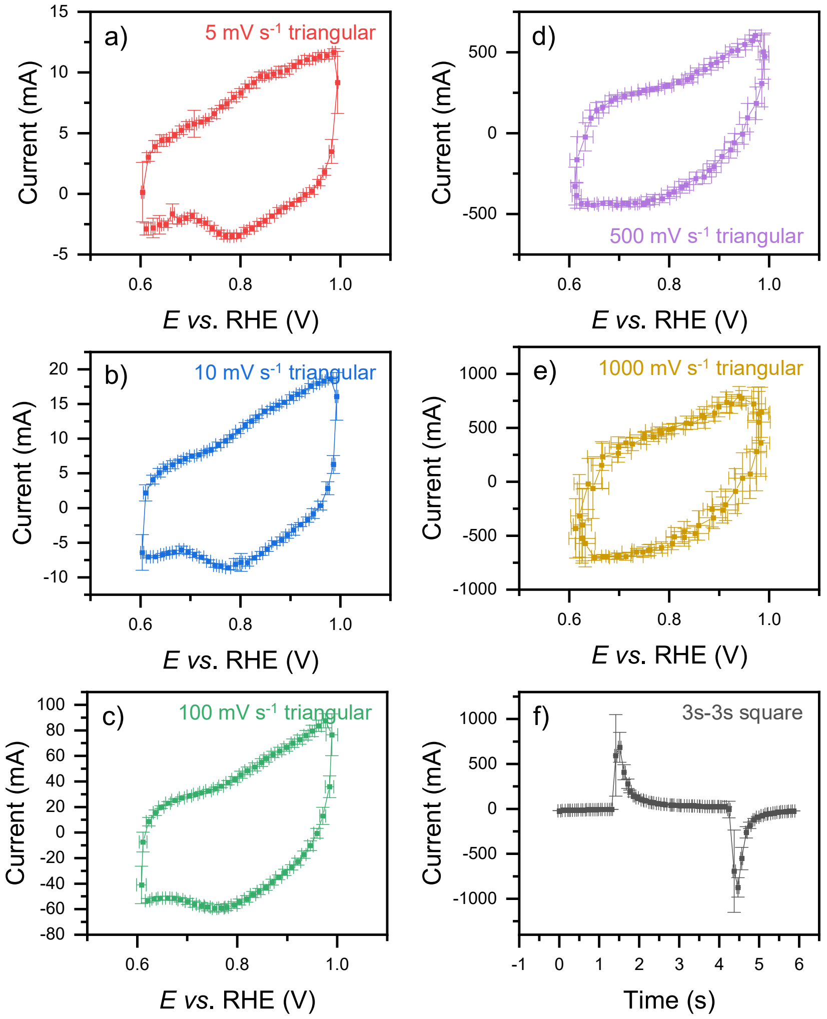

Figure S13: Reconstructed cyclic voltammograms during (ultra-fast) *operando* WAXS measurements in PEMFC. The error bars represent the standard deviations associated to the averaged values during cycle merging. Errors bars correspond to the standard deviations associated with the averaging process from the stroboscopic approach.

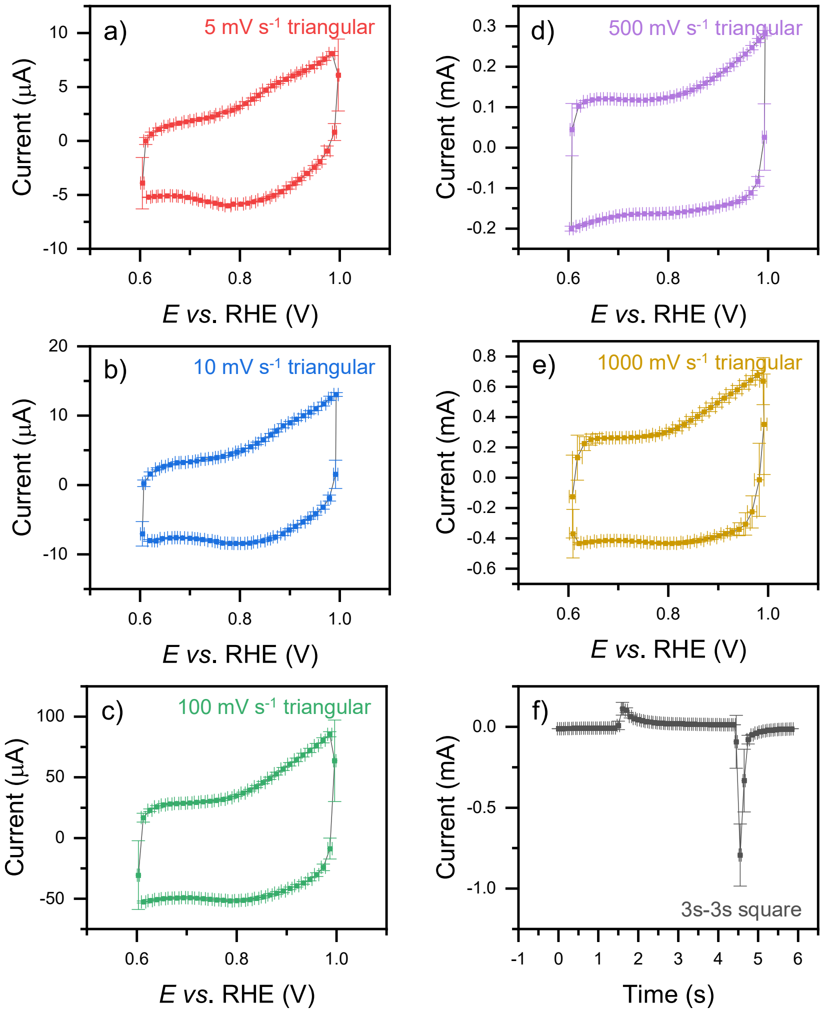

Figure S14: Reconstructed cyclic voltammograms during (ultra-fast) *operando* WAXS measurements in thin-film flow cell. The error bars represent the standard deviations associated to the averaged values during cycle merging. Errors bars correspond to the standard deviations associated with the averaging process from the stroboscopic approach.

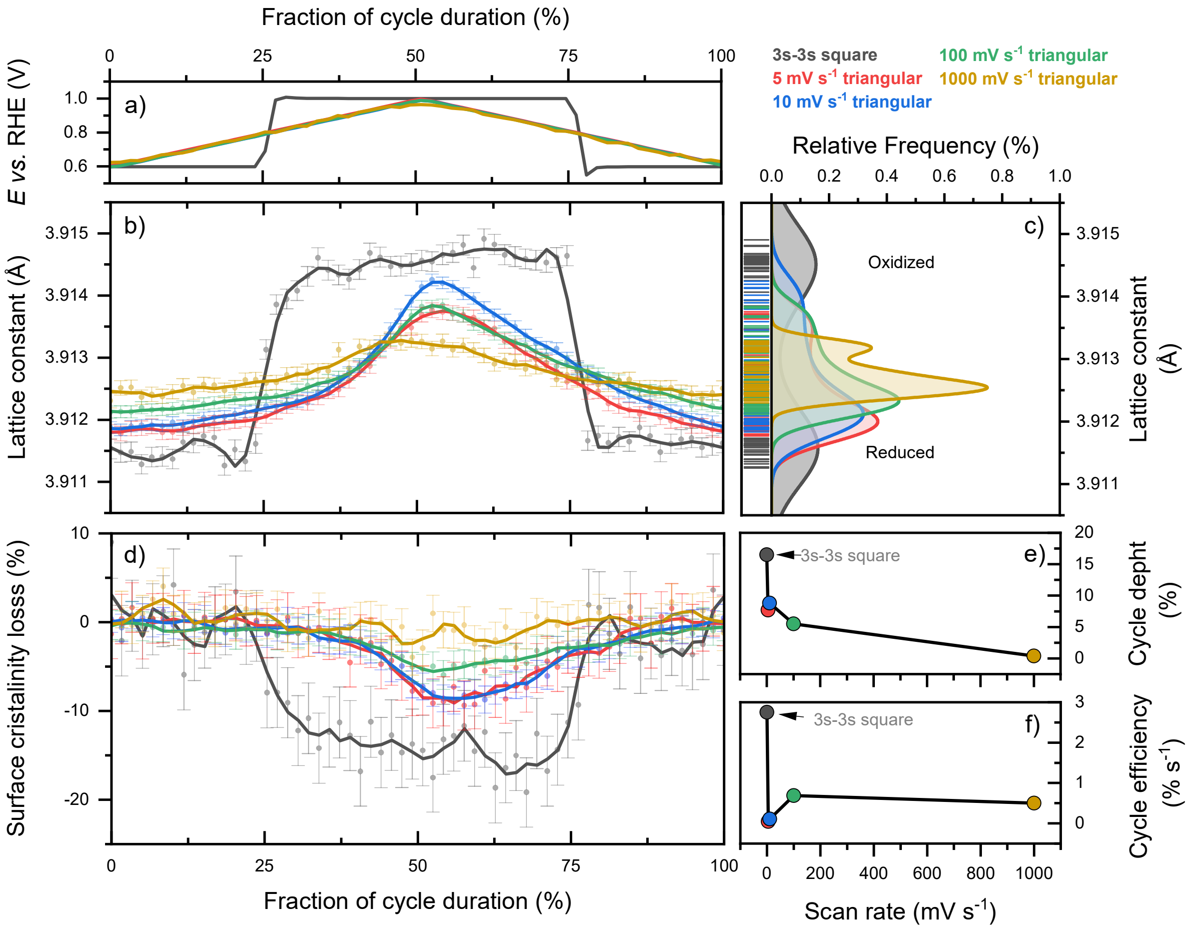

Figure S15: Outpacing Pt oxidation in thin-film catalyst layer during accelerated stress tests. a) Reconstructions of the applied potential cycle profiles between 0.60 and 1.0 V vs. RHE: 3 s – 3 s square wave and triangular waves at 5, 10, 100, 500 and 1000 mV s^{-1}, b) lattice constant variations and c) surface-fraction-normalized scattering intensity variations as a function of the percentage fraction of cycle duration. d) Kernel density estimations of the lattice constant distributions during each potential cycle profile. c) Maximum of surface crystallinity loss induced by a single potential cycle of each profile (cycle depth) and f) amount of surface crystallinity loss induced by each potential cycle profile (cycle efficiency). The voltage was dynamically corrected by the cell high frequency resistance. Errors bars correspond to the standard deviations associated with the refinement of the different parameters.

Table S1: Results from the fits associated with the surface crystallinity loss or recovery during oxidation and reduction in both PEMFC and thin-film electrochemical cells

	y_0 (arb.units)	x_0 (s)	A_1 (arb.units)	t_1 (s)	A_2 (arb.units)	t_2 (s)
**Oxidation PEMFC 80 ºC**	-82 ± 0	1.304 ± 0	48.06 ± 0	0.30 ± 0.02	42 ± 0	3.50 ± 0.2
**Reduction PEMFC 80 ºC**	0.03 ± 0.7	4.24 ± 0	-64.8 ± 2.4	0.31 ± 0.02	n.a.	n.a.
**Oxidation 0.1 M $HClO_4$ 25 ºC**	-8 ± 0	1.509 ± 0	5.6 ± 1.2	0.08 ± 0.014	2.5 ± 0.22	1.15 ± 0.5
**Reduction 0.1 M $HClO_4$ 25 ºC**	0.11 ± 0.07	4.45 ± 0	-7.8 ± 0.36	0.13 ± 0.013	n.a.	n.a.

Table S2: Estimation of the '*RC'* time constant for the two electrochemical cells. C_{DL} is estimated from linear regression of the capacitive current at 0.4 V vs. RHE at different sweep rates and R_s from electrochemical impedance spectroscopy.

	C_{DL}	R_s	**'*RC'* constant**
**PEMFC**	510 mC	0.6 Ω	306 ms
**Thin-film flow cell**	303 µC	24 Ω	7.3 ms

SUPPLEMENTARY REFERENCES

1. Martens, I. *et al.* X-ray transparent proton-exchange membrane fuel cell design for in situ wide and small angle scattering tomography. *J. Power Sources* **437**, 226906 (2019).
2. Martens, I. *et al.* Imaging Heterogeneous Electrocatalyst Stability and Decoupling Degradation Mechanisms in Operating Hydrogen Fuel Cells. *ACS Energy Lett.* **6**, 2742–2749 (2021).
3. Martens, I., Chattot, R. & Drnec, J. Decoupling catalyst aggregation, ripening, and coalescence processes inside operating fuel cells. *J. Power Sources* **521**, 230851 (2022).
4. Chattot, R. *et al.* Beware of cyclic voltammetry! Measurement artefact in accelerated stress test of fuel cell cathode revealed by operando X-ray diffraction. *J. Power Sources* **555**, 232345 (2023).
5. Chattot, R. *et al.* Electrochemical Strain Dynamics in Noble Metal Nanocatalysts. *J. Am. Chem. Soc.* **143**, 17068–17078 (2021).
6. Chattot, R. *et al.* Break-In Bad: On the Conditioning of Fuel Cell Nanoalloy Catalysts. *ACS Catal.* **12**, 15675–15685 (2022).
7. Ashiotis, G. *et al.* The fast azimuthal integration Python library: PyFAI. *J. Appl. Crystallogr.* **48**, 510–519 (2015).
8. Thompson, P., Cox, D. E. & Hastings, J. B. Rietveld Refinement of Debye-Scherrer Synchrotron X-ray Data from A1203. *J. Appl. Crystallogr.* **20**, 79–83 (1987).
9. Montejano-Carrizales, J. M. & Morán-López, J. L. Geometrical characteristics of compact

nanoclusters. *Nanostructured Mater.* **1**, 397–409 (1992).

10. Montejano-Carrizales, J. M., Aguilera-Granja, F. & Morán-López, J. L. Direct Enumeration of the Geometrical Characteristics of Clusters. *Nanostructured Mater.* **8**, 269–287 (1997).
11. Juhás, P., Davis, T., Farrow, C. L. & Billinge, S. J. L. PDFgetX3 : a rapid and highly automatable program for processing powder diffraction data into total scattering pair distribution functions. *J. Appl. Crystallogr.* **46**, 560–566 (2013).
12. Farrow, C. L. *et al.* PDFfit2 and PDFgui: Computer programs for studying nanostructure in crystals. *J. Phys. Condens. Matter* **19**, 0–7 (2007).
13. Kresse, G. & Furthmüller, J. Efficiency of ab-initio total energy calculations for metals and semiconductors using a plane-wave basis set. *Comput. Mater. Sci.* **6**, 15–50 (1996).
14. Kresse, G. & Furthmüller, J. Efficient iterative schemes for *ab initio* total-energy calculations using a plane-wave basis set. *Phys. Rev. B* **54**, 11169–11186 (1996).
15. Perdew, J. P., Burke, K., Ernzerhof, M. & Ernzerhof, M. Generalized gradient approximation made simple. *Phys. Rev. Lett.* **77**, 3865 (1996).
16. Kresse, G. & Joubert, D. From ultrasoft pseudopotentials to the projector augmented-wave method. *Phys. Rev. B* **59**, 1758–1775 (1999).
17. Vogel, W. X-Ray Diffraction from Clusters. *Cryst. Res. Technol* **33**, 1141–1154 (1998).
18. Brown, P. J., Fox, A. G., Maslen, E. N., O'Keefe, M. A. & Willis, B. T. M. Intensity of diffracted intensities. in *International Tables for Crystallography* vol. C 554–595 (International Union of Crystallography, 2006).
